# Generosity among the Ik of Uganda

**DOI:** 10.1017/ehs.2020.22

**Published:** 2020-05-14

**Authors:** Cathryn Townsend, Athena Aktipis, Daniel Balliet, Lee Cronk

**Affiliations:** 1 Department of Anthropology, Baylor University, Waco, TX 76798, USA; 2 Department of Psychology, Arizona State University, Tempe, AZ 85287, USA; 3 Department of Experimental and Applied Psychology, Vrije Universiteit Amsterdam, Amsterdam, The Netherlands; 4 Department of Anthropology and Center for Human Evolutionary Studies, Rutgers, the State University of New Jersey, New Brunswick, NJ 08901, USA

**Keywords:** dictator game, sharing, scarcity, famine, generosity, cooperation

## Abstract

According to Turnbull's 1972 ethnography *The Mountain People*, the Ik of Uganda had a culture of selfishness that made them uncooperative. His claims contrast with two widely accepted principles in evolutionary biology, that humans cooperate on larger scales than other species and that culture is an important facilitator of such cooperation. We use recently collected data to examine Ik culture and its influence on Ik behaviour. Turnbull's observations of selfishness were not necessarily inaccurate but they occurred during a severe famine. Cooperation re-emerged when people once again had enough resources to share. Accordingly, Ik donations in unframed Dictator Games are on par with average donations in Dictator Games played by people around the world. Furthermore, Ik culture includes traits that encourage sharing with those in need and a belief in supernatural punishment of selfishness. When these traits are used to frame Dictator Games, the average amounts given by Ik players increase. Turnbull's claim that the Ik have a culture of selfishness can be rejected. Cooperative norms are resilient, and the consensus among scholars that humans are remarkably cooperative and that human cooperation is supported by culture can remain intact.

## Graphical abstract

**Figure:**
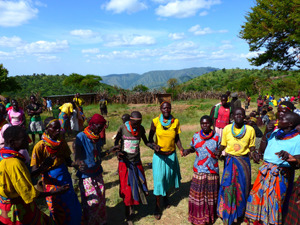

**Media summary:** New research corrects myth about ‘The Mountain People’ (the Ik), infamous for their allegedly selfish and loveless culture.

## Introduction

In the evolutionary biological study of cooperation, it is widely accepted that humans cooperate with each other in a wide variety of ways and on large scales (Herrmann, Thoni, & Gachter, 2008; Cronk & Leech, 2013; Peysakhovich, Nowak, & Rand, 2014; Ensminger & Henrich, 2014; Turchin, 2015; Gächter & Schulz, 2016; Falk et al., 2018) and that culture, defined as socially transmitted information (Cronk 1999; Alvard 2003), is a primary reason for our species’ success in the arena of cooperation (Boyd & Richerson 2009). Those claims contrast sharply with findings presented in *The Mountain People*, an ethnography of the Ik people of northeastern Uganda by Colin Turnbull published in 1972 (Turnbull 1972). According to Turnbull, the Ik he observed in the 1960s were extremely selfish and uncooperative as the result of a cultural adaptation to conditions of scarcity. If Turnbull's claims regarding the selfish nature of Ik culture and its impact on Ik behaviour are valid, then this presents a serious challenge to the present-day consensus regarding human culture and human cooperation. We examine Turnbull's claims, Ik culture and Ik behaviour in an effort to shed light on the relationship between culture and cooperation more broadly.

Turnbull conducted the fieldwork upon which *The Mountain People* is based between 1964 and 1967. According to Turnbull, the Ik had ‘cultivated individualism to its apex’, making them ‘the loveless people’, ‘unfriendly’, ‘uncharitable’ and ‘mean’. He also described many specific examples of such behaviour, including child abandonment. Turnbull found Ik behaviour and Ik culture so repugnant that he advocated that they be forcibly ‘rounded up in something approaching a military operation’ without regard to ‘age, sex, or kinship’ ‘in small units of about ten’ and then ‘taken to parts of Uganda sufficiently remote for them not to be able to return’ to their home as a way of eliminating the culture traits, including their language, that he believed led them to be so selfish.

Unlike most ethnographies, *The Mountain People* was reviewed in such popular publications as *Time* magazine and *The New York Times* and was the subject of an essay in Lewis Thomas’ best-selling collection of essays *The Lives of a Cell (1978)*. As a result, Turnbull's description of the Ik reached a broad general audience and was even the basis of a play performed by the Royal Shakespeare Company that also toured the United States (Higgins & Cannan, 1984). Although Turnbull's claims regarding the Ik were less widely accepted among anthropologists and other scholars than they were among the public (Barth, 1974; Dirks, 1980; Grinker, 2000; Willerslev & Meinert, 2017), *The Mountain People* has been cited frequently, and many scholars have accepted its findings more or less at face value. For example, Margaret Mead praised it as a ‘beautiful and terrifying book of a people who have become monstrous beyond belief’. Ashley Montagu described it as an ‘important book’ about ‘a people who are dying because they have abandoned their humanity’. Carleton Coon described it as ‘a masterpiece’ and as ‘a magnificent if ghastly tale.’ (These quotes are from the book's dust jacket.) In an effort to make the case for the importance of culture in explaining human behaviour, evolutionary biologist Richard Dawkins (1976) used the ‘utter selfishness’ of the Ik as an example of the ‘formidable challenge of explaining culture, cultural evolution, and the immense differences between human cultures around the world’ (p. 205). More recently, sociologist Nicholas A. John (2017), citing *The Mountain People* as ‘an anthropological classic’, described the Ik as living ‘in a state of distrust and discord’ in an effort to undermine the idea that hunter–gatherer societies are typified by widespread sharing (p. 93).

Perhaps owing to the remoteness of Ikland and frequent security problems there, fieldwork-based challenges to Turnbull's claims have been few. The most significant such challenge to date was put forward by linguist Bernd Heine (1985), who conducted fieldwork among the Ik in February and March of 1983. Heine described a variety of serious shortcomings of Turnbull's methods and a large number of inaccuracies in his descriptions of Ik culture and behaviour. Heine observed that ‘Turnbull's account of Ik culture turned out to be at variance with most observations we made – to the extent that at times I was under the impression that I was dealing with an entirely different people’ (p. 3).

The Ik of today live mostly in Ik County in the mountains of northeastern Uganda near the borders with Kenya and South Sudan. Ik County falls under the political administration of the Kaabong district of Karamoja, the poorest region in Uganda. According to the Uganda Bureau of Statistics (2017), Ik County has a total population of 4023 people. There are also Ik living elsewhere in Uganda and in South Sudan and Kenya. The Ik language, which they call *Icé-tód*, is a member of the Kuliak subgroup of the Nilo-Saharan language family (Schrock 2017), which distinguishes them from neighbouring pastoralists.

According to Turnbull, the Ik were mobile hunter–gatherers until the early twentieth century, when they were forced to sedentarize in their current mountain range owing to encapsulation by outsiders. Today, they subsist through a combination of horticulture, beekeeping, hunting and gathering. Ik County has villages that range in size from approximately 50 to approximately 250 people. Owing to the threat of raids by pastoralists, Ik villages are surrounded by wooden palisades with passages that allow villagers to escape down the mountainside.

Contrary to Turnbull's account, we find that Ik culture has many norms that enhance cooperation and encourage generosity. For example, *tomora maráŋ* is an Ik adage meaning ‘it's good to share’. The Ik verb for share, *tomor*, is typically defined by the Ik as to give to someone who is in need (see Table S2). For example, one young man explained that *tomor* is that ‘when you get something, you give to those who are in need’. Food, drink, land, clothing, tools, labour, news, companionship, discoveries and love are all things that Ik people say are appropriate to share. According to Ik interviewees, less pleasant aspects of life, such as trouble and mourning the dead (and the associated material costs), should also be shared. Sharing is particularly important during the dry season, which the Ik refer to as the ‘hunger season’ (*ɲɛ`ƙɛ` tso´i´*). During that time, their fields are unproductive. They rely purely on stored foods and foraging within a territory limited by the threat of attacks by pastoralists. Sharing is also important for survival during intermittent and localized droughts, which cause crop failures and waterborne diseases.

Sharing with those in need is also encouraged by Ik religious beliefs. In their view, the landscape is inhabited by spirits known as *kíʝáwik^a^.* The literal meaning of *kíʝáwik^a^* is ‘children of the Earth’, and they are described as spirits with a shadowy human form. Beliefs in these spirits are idiosyncratic, and some Ik individuals describe them using the English word ‘satanic’. While religious affiliations differ, many Ik have a syncretic cosmology that incorporates beliefs from Christianity and indigenous belief in a creator or supreme god (*didiɡwari´*) alongside capricious nature spirits (*ɲɛki´pyɛ´)* including the *kíʝáwik^a^*. Importantly, *kíʝáwik^a^* are believed to bring misfortune to individuals who fail to share with others and to reward those who are especially generous.

## Methods

The primary methods used were Dictator Games and participant-observation fieldwork among the Ik community of Timu Parish, Ik County, Uganda. To assess Ik generosity and selfishness in a way that would be comparable with similar studies that have been conducted in a wide variety of societies around the world (Marlowe, 2004; Wiessner, 2009; Engel, 2011; Henrich et al., 2014), we conducted a series of unframed and framed Dictator Games. The total sample of Dictator Game participants consists of 120 Ik adults, 55 women and 65 men. Young adults dominate our Ik Dictator Game sample (42 out of 120, or 35% were aged between 18 and 25) and the average age is 34.3 years (SD = 13.9). This is because the population of Ik County is relatively young: 56% are aged 0–17 and 22.9% are 18–30 (Uganda Bureau of Statistics 2017). The dataset for the Dictator Games that we conducted with the Ik is provided in the Supplementary Information (Table S3). Parties interested in the dataset used in the meta-analysis of Dictator Games (Engel, 2011) may contact Christoph Engel at engel@coll.mpg.de.

The research was conducted with permission from, and in compliance with, the Institutional Review Board of Rutgers, the State University of New Jersey and the Uganda National Council for Science and Technology.

The Dictator Games were conducted between January and December 2016, plus return trips in June to July 2017 and November to December 2018 by the lead author, Townsend. Cronk and Aktipis also made a brief visit to the area in 2016. Townsend collected data on Ik demography, residential mobility, kinship, marriage and reproduction, and conducted interviews on the topics of risk, need, sharing and generosity. She also conducted 20 in-depth life history interviews with Dictator Game participants. All research surveys and interviews were conducted in Icé-tód through an Ik interpreter fluent in both Icé-tód and English.

In a standard Dictator Game, a player is given an endowment of money and the opportunity to share some, none or all of it with an anonymous person in their community. The Dictator Game is sometimes described as assessing generosity (Kahneman, Knetsch, & Thaler, 1986; List, 2007; Engel, 2011; Sorokowski et al., 2017). It has also been described as measuring whether players choose, in their decision making, to conform ‘to the selfishness axiom’, which is ‘the assumption that individuals seek to maximize their own material gains in these interactions and expect others to do the same’ (Henrich et al. 2004). The assumption of ‘rational’ self-interest, which lies at the core of classic economic theory, is represented in the Dictator Game by a decision to share none of the allocated money. While decisions that contradict the assumption have been interpreted as motivated by either ‘altruism’ or ‘fairness’ (Guala and Mittone, 2010; Ensminger & Henrich, 2014), both describe possible motivations for behaviour that is other-regarding in the willingness to give (i.e. generous) rather than purely self-regarding (i.e. selfish).

Participants were assigned to play either an unframed Dictator Game (i.e. a control condition) or games that were framed according to culture traits that were observed during the qualitative phase of our research: (a) the ethical principle *tomora maráŋ* that it is good to share with those in need (i.e. the needy recipient condition); and (b) a belief in supernatural punishment of those who fail to share by *kíʝáwik^a^* spirits (i.e. the supernatural punishment condition). We also combined the two framing conditions (i.e. needy recipient and supernatural punishment) to create a fourth framing condition. In all conditions, we provided each player with 2000 Uganda shillings (approximately US$0.50), which is roughly equivalent to one day's wage in their area, in the form of twenty 100 shilling coins. Our first hypothesis was that Dictator Games that include specific conditions that invoke the most important Ik culture traits of generosity would lead to significantly increased donations by Ik participants in these Dictator Games (Hypothesis 1). Our second hypothesis was that Ik behaviour in Dictator Games would be similar to and comparable with studies using the Dictator Game in other societies (Hypothesis 2). For both hypotheses, statistical significance was set at *p* < 0.05.

## Dictator Game Protocol

Participants for the Dictator Games were recruited via convenience sampling. Any adults present in the village at the time of Townsend's visit were invited to participate, and the informed consent procedure was described to them. Following this, subjects were read short instructions about the game that varied depending on condition.

Control:

You will be given 2000 shillings to decide how to share between yourself and another member of your community. You will not find out who this person is, and they will not find out who you are.

Needy recipient:

You will be given 2000 shillings to decide how to share between yourself and another member of your community who is in need. For example, they may be old and hungry. You will not find out who this person is, and they will not find out who you are.

Supernatural punishment:

What do you know of the Earth spirits (*kíʝáwik^a^*)? Do they cause trouble for people who do not share with others? Do they bring good fortune to those who do share with others?

You will be given 2000 shillings to decide how to share between yourself and another member of your community. You will not find out who this person is, and they will not find out who you are.

Needy recipient + supernatural punishment combined:

What do you know of the Earth spirits (*kíʝáwik^a^*)? Do they cause trouble for people who do not share with others? Do they bring good fortune to those who do share with others?

You will be given 2000 shillings to decide how to share between yourself and another member of your community who is in need. For example, they may be old and hungry. You will not find out who this person is, and they will not find out who you are.

The participant was shown twenty 100 shilling coins spread out on a clipboard. If necessary, they were prompted to indicate how they wished to allocate the coins by rearranging the coins on the clipboard. The decisions were recorded. Money that was allocated to another person by a participant was redistributed on a different day, either randomly to any other Ik individual, or randomly to an elderly Ik individual, depending on the condition.

## Results

### Ethnographic observations

In keeping with the culture traits of *tomora maráŋ* and *kíʝáwik^a^* (described in the Introduction), the lead author observed frequent sharing of food and other resources among the Ik. She observed sharing between households occurring on a day-to-day basis, and also experienced a willingness on the part of Ik people to share food and water with her. For example, Ik people often gave her produce from their gardens or bushmeat. Sharing between households is customary year-round and includes food, water, home-brewed maize beer, clothing and money. It is often, but not always, based on need and ties of friendship, kinship or clan affiliation. The dry season involves falling back on foraging for subsistence once the granaries have been depleted. Ik interviewees recognize that this is a time when they could run into life-threatening needs in which they must rely on the willingness of others to share, and therefore cultivating sharing relationships is important. One middle-aged Ik man said, ‘If you keep everything in your granary to yourself and don't share, you will still run out of food during the hunger season, and then you will suffer because no-one will share with you’. When asked what the Ik word for generosity, *batanon*, means to them, six out of 10 interviewees mentioned giving to those in some kind of need (*bedes*). When asked what the Ik word for sharing, *tomor*, means to them, five out of 10 interviewees mentioned giving to those in need (Tables S1, S2).

The culture trait of supernatural punishment (or the moralistic capacity of *kíʝáwik^a^* spirits) was described to Townsend in casual conversations during the course of participant observation. The *kíʝáwik^a^* are portrayed as potentially punishing or rewarding depending on the sharing behaviour of people. During two conditions of the Dictator Game experiments, a total of 60 participants were asked ‘What do you know of the Earth spirits (*kíʝáwik^a^*)? Do they cause trouble for people who do not share with others? Do they bring good fortune to those who do share with others?’ For the ‘do they cause trouble’ question, 46 participants (76.67%) answered in the affirmative. For the ‘do they bring good fortune’ question, 28 participants (46.67%) answered in the affirmative (Table S3). Beliefs about the moralistic capacities of these nature spirits are thus idiosyncratic, with stronger accordance in the case of belief in their punishing capacity.

Ik culture also includes several other traits that encourage generosity and cooperation. For example, the Ik have a culturally instantiated obligation to assist others should they be in need of a new place to live. They consider it especially important to extend such help to friends and to members of one's father's clan, one's mother's clan and the clans of one's in-laws; however, any Ik person who is in serious need of a home should be taken in. We have even documented an instance in which the Ik community permanently accepted two Turkana refugees – a widow and her infant daughter who had lost their family owing to disease and cattle raiding – into their community. The Turkana are a neighbouring and frequently hostile group of pastoralists living in northwestern Kenya. Owing to the problems of attacks from Turkana and other non-Ik pastoralist groups, aid with relocations sometimes also occurs on the scale of entire villages. As a result, several Ik villages were previously located elsewhere. For example, the village of Loodoi–Lokinene is the result of a fusion of two previously distinct villages that occurred when the Lokinene villagers were forced to flee because of recurrent attacks.

### Hypothesis testing

#### Hypothesis 1

**Figure 1.**
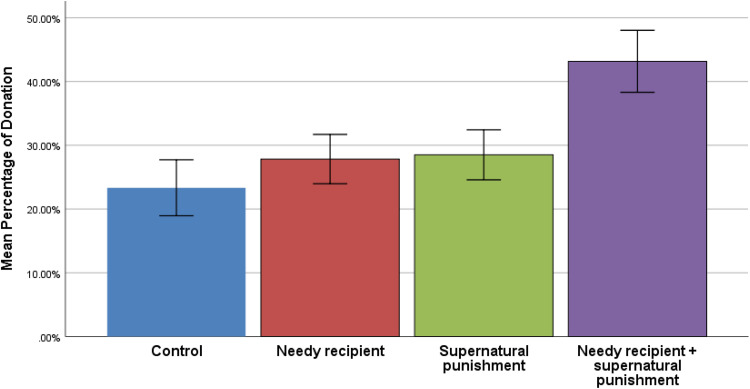
Mean percentage donated in the four conditions of the Dictator Game. Participants in the combined game donated significantly more than participants in the control game. *N* = 30 participants per game; error bars represent SE, statistical significance was determined via Kruskal–Wallis *H*, followed by Dunn's multiple comparison (*p* < 0.01).

**Figure 2.**
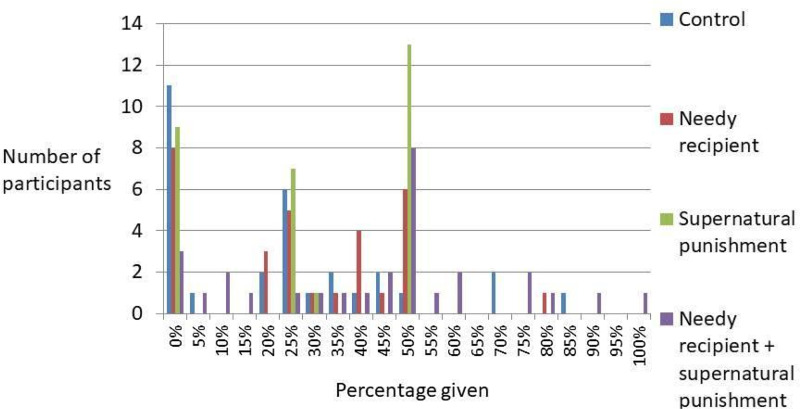
Distribution of proportions donated across the four conditions of the Dictator Game.

The descriptive statistics of the Dictator Games played among Ik participants are displayed in Figures 1 and 2, and in Table 1. The combined mean generosity of the Ik in Dictator Game experiments was calculated by including all four conditions: *n* = 120, mean (*M*) = 0.302, standard error (SE) = 0.022, median = 0.25, mode = 0, standard deviation (SD) = 0.246.

**Table 1.** Descriptive statistics of each of the four conditions of the Ik Dictator Games

	Control	NR	Pun	NR + Pun
N	30	30	30	30
Mean	0.233	0.253	0.29	0.432
SE	0.044	0.040	0.039	0.049
Median	0.25	0.25	0.275	0.5
Mode	0	0	0.5	0.5
SD	0.24	0.221	0.216	0.266
Range	0.85	0.8	0.5	1
Minimum	0	0	0	0
Maximum	0.85	0.8	0.5	1

NR, Needy recipient; Pun, supernatural punishment; NR + Pun, needy recipient combined with supernatural punishment.

We conducted a multivariate regression analysis to predict generosity in the Dictator Game. In the model, we include both participant age and gender, and then include three dummy variables that we compare with the control conditions: (a) the needy recipient frame; (b) the supernatural punishment frame; and (c) the combined frame (needy recipient + supernatural punishment). The model is reported in Table 2. Age and gender were not associated with generosity in the Dictator Game. We found no significant differences between the control condition and the needy recipient condition or the control condition and the supernatural punishment condition. However, participants were more generous in the combined frame condition relative to the control conditions (*β* = 0.346, *p* = 0.002). Hypothesis 1 is thus partially supported by our data.

**Table 2.** Multivariate regression analysis predicting generosity in the Dictator Game

	*β*	*t*	*p*
Age	0.012	0.134	0.893
Gender	0.123	1.38	0.169
NR	0.089	0.818	0.415
Pun	0.086	0.789	0.432
NR + Pun	0.346	3.17	0.002

Participants were sampled across 18 villages. We also ran the model described above (and displayed in Table 2) with two multilevel models that (a) only allowed the intercept to vary across villages (see Table S4) or (b) allowed both the intercept and slopes to vary across villages (see Table S5). Both of these models resulted in the same conclusion of the simple regression model that does not account for any variance in the results that might have occurred across villages (see Table S6 for syntax for the two analyses in SPSPP).

#### Hypothesis 2

In order to test our hypothesis that Ik players in the Dictator Game would behave similarly to players from other societies, we compared the results in a cross-cultural meta-analysis using previous studies that used Dictator Games with similar protocols (Marlowe, 2004; Wiessner, 2009; Ensminger & Henrich, 2014; see Henrich et al., 2014 for a summary of results; see Table S7 for all data in the cross-cultural meta-analysis).

Previous studies suggest that transfers in economic games tend to be lower in societies with only marginal integration into the global market economy compared with societies that are highly integrated (Tracer, 2004; Henrich et al., 2010; Ensminger & Henrich, 2014; Sorokowski et al., 2017). The societies of the Ju/’hoansi (Wiessner, 2009) and the Hadza (Marlowe, 2004) are similar to that of the Ik in that they are all African societies that have a recent history in which hunting and gathering were dominant, and their incorporation into the market economy is marginal.

The studies reported in Ensminger and Henrich (2014), Wiessner (2009) and Marlowe (2004) observed giving a percentage of an endowment in a Dictator Game to another anonymous person, and are therefore most comparable with our control treatment (i.e. the unframed Dictator Games). We conducted a random effects meta-analysis, using Comprehensive Meta-analysis software version 3, of the percentage of the endowment given in the Dictator Game across these 17 studies and then calculated a prediction interval around that point estimate (using the formulae reported in Borenstein, Hedges, Higgins, & Rothstein, 2011).

**Figure 3.**
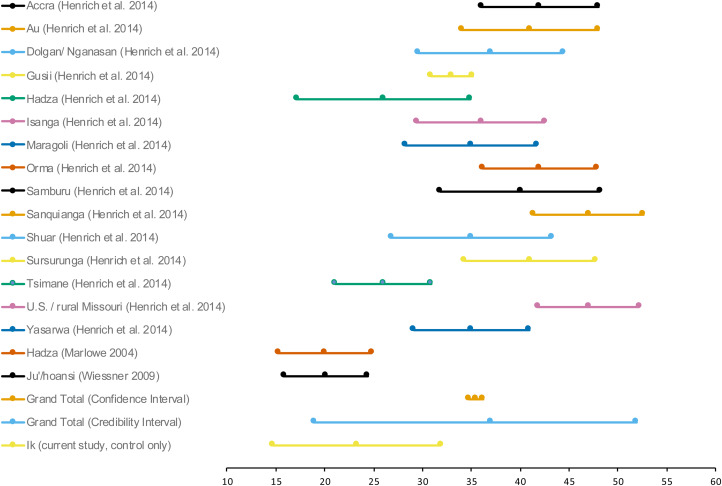
Studies included in the cross-cultural meta-analysis of generosity in the Dictator Game.

Based on the observed data, the prediction interval reports the range of predicted true values for the next observation (Higgins, Thompson, & Spiegelhalter, 2009). Here we can treat the outcome of our study at the next observation and observe whether the outcome fits inside the prediction interval. Across these 17 studies, a random effects meta-analysis indicates an average amount of 35.4% of endowment given in the Dictator Game (*M* = 0.354, 95% Confidence Interval [CI] Lower Limit [LI] = 0.313, Upper Limit [Upper Limit] = 0.395). Calculating the prediction interval (see Borenstein et al., 2011), we find that, based on the observed data in these 17 studies, the range of predicted true scores for the next study can fall between giving 18.9 and 51.9% of the endowment in the Dictator Game (*k* = 17, *M* = 0.37, 95% Prediction Interval [PI] LL = 0.189, UL = 0.519). In the control condition, we observed that the Ik gave 23.33% of their endowment to an anonymous other, which fits within the prediction, i.e. credibility, interval (depicted in Figure 3).

As a further test of Hypothesis 2, we compared Ik Dictator Game play with that of players in a large meta-analysis of Dictator Game experiments (Engel, 2011). Out of a total of 21,258 participants in these experiments, 19,669 were from Western countries (92.5%). Because not all studies included in the meta-analysis reported the standard error, Engel used a method to estimate it. Here, we consider only the studies that reported the standard error (*k* = 445). Several of these estimates come from the same study, such as different treatments within a study or paper, and so are not independent observations. Therefore, we conducted a multi-level random effects meta-regression using R package metafor (Viechtbauer, 2010), which also calculates the prediction interval.

Replicating the analysis reported by Engel (2011), we found that across these studies people give on average 27.7% of their endowment in the Dictator Game (*k* = 445; *M* = 0.277, 95% CI LL = 0.24, UL = 0.31). Based on the studies, the expected range of true values of the next study is an average of 9.7–46.00% of endowments in the Dictator Game (95% CI LL = 0.097, UL = 0.46). Because the studies reported by Engel (2011) include a variety of experimental treatments, the most appropriate comparison is with the average generosity observed in the Ik sample across all four treatments. Overall, the Ik gave a grand mean of 30.71% of their endowment across the treatments. This fits within the credibility interval, suggesting that, given the amounts of generosity we observed in this range of studies, we could expect what we found in the Ik data.

## Discussion

Across our analyses of Dictator Games, considering what we know about generosity in a range of different societies, the generosity observed among the Ik falls within the expected range of possible giving. These analyses suggest that the Ik are not outliers in their average amounts of giving in Dictator Games. Our experiment also shows a statistically significant difference between generosity in the condition with both the needy recipient and supernatural punishment condition when compared with the control condition, but the difference is not statistically significant when comparing the control condition with either needy recipient or supernatural priming alone. We suspect that this reflects the real-world cultural environment of Ik participants in which the culture traits of *tomora maráŋ* and belief in *kíʝáwik^a^* interact with one another to encourage generosity. Some caution is required owing to the small sample size and convenience sampling strategy. However, our participant observation also indicates that Ik culture encourages generosity. Together these results suggest that scholars should desist from using the Ik as an example of how culture can make people behave selfishly. In addition, the consensus among scholars that humans are remarkably cooperative and that human cooperation is supported by culture can remain intact despite Turnbull's account of the Ik.

Dictator Game experiments are mostly conducted among student populations in Western countries, who may behave less generously in the experiment than older adults (Engel, 2011; Ensminger & Cook, 2014). A high proportion of the Dictator Games in the second meta-analysis (Engel, 2011), used as a comparison for Hypothesis 2, involved student age participants (20,121 out of 21,538, or 94.6%) and 538 participants (2.5%) were children. It shows a linear relationship between age and generosity. In comparison, we did not observe a relationship between age and generosity, and young adults make up a considerable portion of our Dictator Game sample (see Methods).

A belief in some form of supernatural punishment has been hypothesized to be an important incentive for cooperative behaviour across societies (Shariff and Norenzayan, 2007; Atkinson & Bourrat, 2011; Aveyard, 2014; Johnson, 2015; Cronk & Aktipis, 2017). Scholars have argued that supernatural punishment administered by moralizing gods is characteristic of modern world religions and complex hierarchical societies, in which it is thought to facilitate wide-scale cooperation among non-kin (Henrich et al., 2010; Norenzayan et al., 2016; Purzycki et al., 2016; Shariff et al., 2016). Yet moralistic supernatural beliefs are also found, albeit at relatively low rates, among hunter–gatherers (Dickson et al., 2005). There are differing interpretations about whether moralizing gods have been instrumental in creating social complexity, or whether they came about after the emergence of complexity (Lang et al., 2019; cf. Whitehouse et al., 2019; Beheim et al., 2019, cf. Savage et al., 2019). Our study may be relevant to these debates. However, since there has evidently been syncretism between Animism and Christianity, we advise caution on how the Ik culture trait of supernatural punishment is interpreted in relation to social organization and the scale of cooperation.

Wiessner (2009) notes of the Ju/’hoansi that their relatively selfish behaviour in Dictator Games, a context which she describes as ‘a social and cultural vacuum’, contrasts with their behaviour in ordinary life, in which sharing is commonplace. In a similar vein, the anonymous and unframed Dictator Games are alien to the day-to-day context of Ik life, in which visible sharing with associates is socially expected. An additional factor that may influence participants from marginally market-integrated societies to give less in Dictator Games is that cash is a relatively scarce and valuable resource. For example, Ik people often lack the cash needed to obtain medical treatment at a clinic. Cash is associated with transactional behaviour while goods like food and housing are associated with sharing.

In light of Turnbull's claims about Ik selfishness being a product of scarcity, it is important that Ik consider it conventional to share more, not less, during the hunger season. Such sharing during times of scarcity is by no means unique to the Ik. On the contrary, systems of risk-pooling through transfers to those in need are common cross-culturally (Cronk et al., 2019). Computer simulations have shown that such need-based transfers are more effective in both pooling risk and enhancing survival than transfers that are contingent upon repayment, particularly in environments characterized by volatility and unpredictable resource availability (Aktipis, Cronk, & de Aguiar, 2011; Aktipis et al., 2016; Hao, Armbruster, Cronk, & Aktipis, 2014).

What, then, should we make of Turnbull's depiction of the Ik? As is clear from Turnbull's own description, drought and famine during the years 1965–1966 pushed the Ik into a state of physiological and psychological exhaustion. During Turnbull's visit, famine led to the deaths of several Ik children and elders whom he knew. Ik people today remember the year 1966 as a particularly severe famine year. This suggests that the societal collapse witnessed by Turnbull was due not to Ik culture but rather to Ik individuals losing the physical capacity to cooperate because of extreme famine, as anthropologist Robert Dirks (1980) argued in a cross-cultural comparison of behaviour during famines. We conclude that the selfish behaviour that Turnbull observed was a by-product of famine and not a cultural adaptation to conditions of chronic scarcity. An analogy illustrates this logic: there is historical evidence of human flesh consumption during the Irish Potato Famine of the late 1840s (Ó Gráda, 2015), and cannibalism has been documented in many other historical famines around the world (Schutt, 2017). Yet the Irish are not labelled as cannibals and Irish culture has not been labelled cannibalistic (nor should it be). It makes no more sense to use the Ik as an example of how culture encourages selfish behaviour than it would to use the Irish as an example of how culture encourages cannibalism. If the Ik case study can tell us anything about culture, it is that even when conventions of generosity collapse owing to extremely stressful conditions, as reportedly occurred among the Ik, it is possible for them to completely re-emerge within 50 years, as our findings indicate they have.

Turnbull's most fundamental error may have been to assume that all Ik behaviour, along with all human behaviour more broadly, is best explained with reference to culture. Such an assumption is widespread but rarely critically examined or tested (D'Andrade, 1992). The relationship between culture and behaviour is currently not well understood, but one possibility is that we have evolved to be particularly susceptible to conventions that help us coordinate our social behaviours because otherwise we would not be able to reap the benefits of coordination (Cronk, 2017). Computer simulations show that the choice between sharing with those in need with no expectation of repayment and sharing that is contingent upon repayment is a type of coordination problem known as a Stag Hunt (Rousseau, 1992; Skyrms, 2004; Aktipis et al., 2016). Thus, sharing with those in need, as we have seen among the Ik and elsewhere, may be just such a coordination convention with Stag Hunt payoffs, where the benefit from helping each other in times of need accrues only if both individuals are using this strategy.

Since the famine of 1965–1966, the Ik have been through another famine and cholera epidemic in 1981. They experienced a worsening of intergroup hostilities as the result of firearms proliferation in a period of political instability in East Africa from the 1940s to the 1990s, and in particular, hostilities between the Ugandan government and the Lord's Resistance Army, an insurgency operating in northern Uganda and South Sudan during the 1990s and early 2000s. The resilience of Ik culture traits of generosity in the face of such extreme hardships is cause for optimism in the context of the world's refugees and precarious populations living in failed states.

## Data Availability

The data that support the findings of this study are available in the Supplementary Information with the exception of the dataset used in the meta-analysis of Dictator Games (Engel 2011). Restrictions apply to the availability of these data, which were used under licence for this study. Parties interested in this dataset may contact Christoph Engel at engel@coll.mpg.de.

## Supplementary material

For supplementary material accompanying this paper visit http://dx.doi.org/10.1017/ehs.2020.22. click here to view supplementary material
