# Generosity among the Ik of Uganda – Corrigendum

**DOI:** 10.1017/ehs.2021.3

**Published:** 2021-01-14

**Authors:** Cathryn Townsend, Athena Aktipis, Daniel Balliet, Lee Cronk

The authors apologise that a few typographical errors were included in the publication of the above article.

In Table 1, the mean for the NR (i.e. needy recipient) condition is 0.278 (not 0.253), the median for the Pun (i.e. punishment) condition is 0.25 (not 0.275), and the SD for the NR condition is 0.212 (not 0.221). In Figure 2, one person is missing from the column representing the number of people in the nr + pun (i.e. needy recipient plus supernatural punishment) condition who gave away 40% of their endowments. These errors have no impact on the article's findings or conclusions.
