# Supplementary material for: Generosity among the Ik of Uganda
Source: Evol Hum Sci. 2020 May 14;2:e23. doi: 10.1017/ehs.2020.22 (PMC10427480; doi:10.1017/ehs.2020.22)
Supplement: Supplementary file 1 [file ehssup.zip › S2513843X20000225sup001.docx]

**Tables S1, S2**

Showing Interview Responses on the topics of Generosity and Sharing

**Table S1: Generosity (*Batanon)***

| Participant Information | Response when asked what the word *batanon* means to them. |
| --- | --- |
| Young man | - Easiness, simplicity - Someone asks and you give - If you find lost property, you show it in public to find the owner. - Giving assistance to the needy e.g. food or clothing |
| Elder man | - Joy (*ilakason*) - Hands spreading in all directions to people in need without discriminating (the expression for this hands metaphor is *zikibiteide kwetikak*). White people are best at this. - If you are provoked, you take it easy, you don’t get angry. If the person continues, you avoid them by moving in order to avoid being provoked and losing your temper. |
| Elder woman | - Giving people food when they are in need, even if you haven’t prepared for them, and they arrive when you are eating. - Giving to someone who asks; being easy towards others, especially those who ask. - Not just food, you can give anything that is useful. |
| Young man | - Responding positively to someone who asks for assistance, giving what they ask for. - Responding to someone who asks you to do some work. - *Batanon* is also being polite; not rude, not hot-tempered, just easy-going. |
| Young woman | - Giving to the needy (*ngitsani*) e.g. clothes, soap, food for people who are hungry (*neka na bubuae),* drinking water or money. |
| Young woman | - When someone comes to you, you give them what they need if it is there. - If you sense trouble (*mena ni gaan) you take it easy, don’t react (mo imisid).* |
| Young man | - Welcoming people, especially visitors (*waniik*), even if you don’t know them. - Being a person that children like (*ewanitetesa asi wicek).* - When you have food and you see people around you, you give to them. |
| Young man | - Helping one another (*ingaress).* - Supporting your brother when you sense a fall(*rumanak*) in their family. - Giving something from your own family to a needy family. |
| Young man | - Being a good person (*daidad*). - You extend your hand (*zikibiteide kwetikak*). |
| Young woman | - Giving. - You don’t discriminate, you are neutral. - If you are not generous, you will lean on one side / be unbalanced. |

**Table S2: Sharing (*Tomor*)**

| Participant Information | Response when asked what the word *tomor* means to them |
| --- | --- |
| Young man | - The way of distributing things where they are needed to neighbours e.g. food, clothing, work done in groups (e.g. labour in gardens). - Sharing trouble, e.g. mourning and sorrow in cases of death. Mourning is shared in several ways e.g. contribution of labour to dig the grave, contribution to the mourners in the way of food & drinks. - We also share land for cultivation with friends and relatives. |
| Elder man | - Sharing good conversations, like we are doing now. - Sharing food: that person gives, you also give. - You can share with brothers, friends and in-laws. |
| Elder woman | - Putting food in one dish so that people can all take from the same dish. Surrounding one dish. - Sharing snuff. Anyone who asks, gets. |
| Young man | - It is a way of getting together. Something is brought from one family, & then members of other families come and sit around and everyone eats. - It also refers to news. For example, from someone who goes to another place like Kaabong [district town outside of Ikland]. People sit around and share information. - We also share farm tools. Not everyone has their own, so you can lend if you have. - Sharing clothes. If someone’s clothes are very dirty & they want to go into town, he can borrow clean clothes. |
| Young woman | - When you get something, you give to those who are in need. - Giving to neighbours, even without them asking. |
| Young woman | - Giving to your neighbour when they sleep hungry. You give them something to eat and drink. - Sharing clothing if someone is in need & they ask you. Even if they have not asked, you can give so long as they are in need of covering. |
| Young man | - News & conversation. - Food: getting together in one spot to eat. - Planning (*huetes)* for cultivation. Elders & middle-aged people meet to discuss where to cultivate. Clan members decide together where to cultivate each year. - We also share planning for marriages, marriages are clan decisions. The family heads get together for discussion and choosing. |
| Young man | - Giving others things that are useful from your own property. - Stories during the evening, the stories of the day. |
| Young woman | - Anything you get, you share, e.g. things from the bush. - Things from the home are shared e.g. clothing, shoes & salt. - Sharing new discoveries from journeys, life experiences. - Informing & teaching, even if they don’t at first understand. Repetition is necessary. |
| Young man | - Loving one another (*tsamunos)* - Peace in the family. - Telling each other stories & news. - If you get a small thing, you divide it with your brother e.g. meat from the bush. You never know whether he may be suffering, & he could die if you don’t share. |

Table S3.

Dictator game decisions divided into four conditions with different framing.

Key to reading table

Participant: code for individual participant

Condition: 1) control, 2) needy recipient, 3) supernatural punishment, 4) combined needy recipient & supernatural punishment.

Allocation decision in Ugandan shillings: dictator / recipient

Percentage: percentage of endowment given to recipient

Gender: female = 1; male = 2

Age: age of participant in years

Village: code for participant’s village of residence in Ik County

Q2: participant’s response to question two of the priming for supernatural punishment condition (Do they [*kíʝáwik^a^*] cause trouble for people who do not share with others?); yes = 1, no = 0

Q3: participant’s response to question three of the priming for supernatural punishment condition (Do they [*kíʝáwik^a^*] bring good fortune to those who do share with others?); yes = 1, no = 0

| **Participant** | **Condition** | **Allocation** | **%** | **Gender** | **Age** | **Village** | **Q2** | **Q3** |
| --- | --- | --- | --- | --- | --- | --- | --- | --- |
| 1 | 0 | 600 / 1,400 | 70% | 1 | 25 | 1 | n/a | n/a |
| 2 | 0 | 1,600 / 400 | 20% | 1 | 20 | 1 | n/a | n/a |
| 3 | 0 | 1,300 / 700 | 35% | 1 | 76 | 1 | n/a | n/a |
| 4 | 0 | 2,000 / 0 | 0% | 1 | 71 | 1 | n/a | n/a |
| 5 | 0 | 1,400 / 600 | 30% | 2 | 19 | 1 | n/a | n/a |
| 6 | 0 | 1,200 / 800 | 40% | 2 | 18 | 1 | n/a | n/a |
| 7 | 0 | 1,100 / 900 | 45% | 2 | 24 | 2 | n/a | n/a |
| 8 | 0 | 1,500 / 500 | 25% | 2 | 22 | 3 | n/a | n/a |
| 9 | 0 | 1,900 / 100 | 5% | 1 | 27 | 4 | n/a | n/a |
| 10 | 0 | 2,000 / 0 | 0% | 2 | 24 | 5 | n/a | n/a |
| 11 | 0 | 1,500 / 500 | 25% | 2 | 21 | 4 | n/a | n/a |
| 12 | 0 | 2,000 / 0 | 0% | 2 | 70 | 6 | n/a | n/a |
| 13 | 0 | 1,500 / 500 | 25% | 2 | 26 | 4 | n/a | n/a |
| 14 | 0 | 600 / 1,400 | 70% | 2 | 29 | 4 | n/a | n/a |
| 15 | 0 | 2,000 / 0 | 0% | 1 | 22 | 3 | n/a | n/a |
| 16 | 0 | 1,000 / 1,000 | 50% | 1 | 26 | 4 | n/a | n/a |
| 17 | 0 | 2,000 / 0 | 0% | 2 | 22 | 6 | n/a | n/a |
| 18 | 0 | 1,100 / 900 | 45% | 1 | 45 | 4 | n/a | n/a |
| 19 | 0 | 1,300 / 700 | 35% | 1 | 36 | 3 | n/a | n/a |
| 20 | 0 | 300 / 1,700 | 85% | 1 | 38 | 7 | n/a | n/a |
| 21 | 0 | 2,000 / 0 | 0% | 2 | 40 | 7 | n/a | n/a |
| 22 | 0 | 2,000 / 0 | 0% | 2 | 22 | 8 | n/a | n/a |
| 23 | 0 | 1,600 / 400 | 20% | 2 | 29 | 9 | n/a | n/a |
| 24 | 0 | 2,000 / 0 | 0% | 1 | 20 | 9 | n/a | n/a |
| 25 | 0 | 2,000 / 0 | 0% | 1 | 45 | 9 | n/a | n/a |
| 26 | 0 | 2,000 / 0 | 0% | 1 | 25 | 10 | n/a | n/a |
| 27 | 0 | 2,000 / 0 | 0% | 2 | 23 | 10 | n/a | n/a |
| 28 | 0 | 1,500 / 500 | 25% | 1 | 45 | 10 | n/a | n/a |
| 29 | 0 | 1,500 / 500 | 25% | 2 | 44 | 10 | n/a | n/a |
| 30 | 0 | 1,500 / 500 | 25% | 2 | 30 | 6 | n/a | n/a |
|  |  |  |  |  |  |  |  |  |
| 31 | 1 | 2,000 / 0 | 0% | 1 | 20 | 7 | n/a | n/a |
| 32 | 1 | 2,000 / 0 | 0% | 2 | 23 | 7 | n/a | n/a |
| 33 | 1 | 1,500 / 500 | 25% | 2 | 23 | 7 | n/a | n/a |
| 34 | 1 | 1,000 / 1,000 | 50% | 1 | 20 | 7 | n/a | n/a |
| 35 | 1 | 1,200 / 800 | 40% | 2 | 19 | 4 | n/a | n/a |
| 36 | 1 | 1,300 / 700 | 35% | 2 | 48 | 6 | n/a | n/a |
| 37 | 1 | 2,000 / 0 | 0% | 2 | 36 | 6 | n/a | n/a |
| 38 | 1 | 2,000 / 0 | 0% | 2 | 19 | 6 | n/a | n/a |
| 39 | 1 | 1,000 / 1,000 | 50% | 1 | 18 | 3 | n/a | n/a |
| 40 | 1 | 400 / 1,600 | 80% | 2 | 20 | 7 | n/a | n/a |
| 41 | 1 | 2,000 / 0 | 0% | 2 | 20 | 7 | n/a | n/a |
| 42 | 1 | 1,000 / 1,000 | 50% | 1 | 45 | 5 | n/a | n/a |
| 43 | 1 | 1,600 / 400 | 20% | 1 | 26 | 1 | n/a | n/a |
| 44 | 1 | 1,000 / 1,000 | 50% | 1 | 38 | 5 | n/a | n/a |
| 45 | 1 | 1,200 / 800 | 40% | 1 | 21 | 1 | n/a | n/a |
| 46 | 1 | 1,100 / 900 | 45% | 2 | 50 | 4 | n/a | n/a |
| 47 | 1 | 1,200 / 800 | 40% | 1 | 41 | 1 | n/a | n/a |
| 48 | 1 | 2,000 / 0 | 0% | 1 | 20 | 4 | n/a | n/a |
| 49 | 1 | 1,200 / 800 | 40% | 2 | 36 | 10 | n/a | n/a |
| 50 | 1 | 1,400 / 600 | 30% | 1 | 36 | 11 | n/a | n/a |
| 51 | 1 | 1,000 / 1,000 | 50% | 2 | 20 | 12 | n/a | n/a |
| 52 | 1 | 1,000 / 1,000 | 50% | 2 | 28 | 4 | n/a | n/a |
| 53 | 1 | 1,600 / 400 | 20% | 2 | 20 | 5 | n/a | n/a |
| 54 | 1 | 1,500 / 500 | 25% | 2 | 40 | 5 | n/a | n/a |
| 55 | 1 | 1,500 / 500 | 25% | 1 | 27 | 5 | n/a | n/a |
| 56 | 1 | 1,600 / 400 | 20% | 1 | 23 | 11 | n/a | n/a |
| 57 | 1 | 1,500 / 500 | 25% | 1 | 32 | 11 | n/a | n/a |
| 58 | 1 | 1,500 / 500 | 25% | 1 | 31 | 12 | n/a | n/a |
| 59 | 1 | 2,000 / 0 | 0% | 1 | 20 | 5 | n/a | n/a |
| 60 | 1 | 2,000 / 0 | 0% | 1 | 38 | 13 | n/a | n/a |
|  |  |  |  |  |  |  |  |  |
| 61 | 2 | 2,000 / 0 | 0% | 1 | 30 | 1 | 0 | 0 |
| 62 | 2 | 1,000 / 1,000 | 50% | 2 | 51 | 14 | 1 | 1 |
| 63 | 2 | 1,000 / 1,000 | 50% | 2 | 56 | 1 | 1 | 1 |
| 64 | 2 | 1,500 / 500 | 25% | 2 | 26 | 14 | 0 | 0 |
| 65 | 2 | 1,000 / 1,000 | 50% | 2 | 23 | 13 | 1 | 1 |
| 66 | 2 | 2,000 / 0 | 0% | 1 | 38 | 13 | 1 | 0 |
| 67 | 2 | 1,000 / 1,000 | 50% | 1 | 39 | 1 | 1 | 1 |
| 68 | 2 | 1,000 / 1,000 | 50% | 2 | 59 | 3 | 1 | 1 |
| 69 | 2 | 1,000 / 1,000 | 50% | 1 | 28 | 6 | 1 | 0 |
| 70 | 2 | 2,000 / 0 | 0% | 1 | 45 | 14 | 1 | 0 |
| 71 | 2 | 1,500 / 500 | 25% | 1 | 42 | 1 | 1 | 1 |
| 72 | 2 | 1,500 / 500 | 25% | 2 | 45 | 15 | 1 | 0 |
| 73 | 2 | 2,000 / 0 | 0% | 1 | 33 | 6 | 1 | 0 |
| 74 | 2 | 2,000 / 0 | 0% | 2 | 26 | 1 | 0 | 0 |
| 75 | 2 | 2,000 / 0 | 0% | 2 | 42 | 5 | 1 | 0 |
| 76 | 2 | 1,000 / 1,000 | 50% | 2 | 58 | 1 | 1 | 1 |
| 77 | 2 | 1,000 / 1,000 | 50% | 2 | 69 | 16 | 1 | 1 |
| 78 | 2 | 1,500/ 500 | 25% | 1 | 27 | 1 | 1 | 0 |
| 79 | 2 | 1,500/ 500 | 25% | 2 | 30 | 13 | 1 | 0 |
| 80 | 2 | 1,000 / 1,000 | 50% | 2 | 21 | 1 | 1 | 1 |
| 81 | 2 | 2,000 / 0 | 0% | 2 | 80 | 1 | 1 | 0 |
| 82 | 2 | 1,000 / 1,000 | 50% | 2 | 33 | 1 | 1 | 1 |
| 83 | 2 | 1,000 / 1,000 | 50% | 2 | 24 | 3 | 1 | 1 |
| 84 | 2 | 1,500/ 500 | 25% | 2 | 23 | 8 | 1 | 0 |
| 85 | 2 | 1,000 / 1,000 | 50% | 2 | 24 | 7 | 1 | 1 |
| 86 | 2 | 2,000 / 0 | 0% | 1 | 33 | 1 | 0 | 0 |
| 87 | 2 | 1,500 / 500 | 25% | 1 | 35 | 3 | 1 | 0 |
| 88 | 2 | 2,000 / 0 | 0% | 1 | 42 | 1 | 0 | 0 |
| 89 | 2 | 1,000 / 1,000 | 50% | 1 | 38 | 3 | 1 | 1 |
| 90 | 2 | 1,400 / 600 | 30% | 1 | 43 | 5 | 0 | 1 |
|  |  |  |  |  |  |  |  |  |
| 91 | 3 | 1,000 / 1,000 | 50% | 2 | 23 | 9 | 1 | 1 |
| 92 | 3 | 1,000 / 1,000 | 50% | 1 | 30 | 1 | 0 | 0 |
| 93 | 3 | 1,800 / 200 | 10% | 1 | 19 | 1 | 1 | 0 |
| 94 | 3 | 1,900 / 100 | 5% | 1 | 28 | 1 | 0 | 0 |
| 95 | 3 | 500 / 1,500 | 75% | 1 | 55 | 1 | 1 | 1 |
| 96 | 3 | 1,000 / 1,000 | 50% | 1 | 38 | 1 | 1 | 0 |
| 97 | 3 | 1,000 / 1,000 | 50% | 1 | 45 | 1 | 1 | 1 |
| 98 | 3 | 1,800 / 200 | 10% | 1 | 19 | 1 | 1 | 0 |
| 99 | 3 | 1,400 / 600 | 30% | 1 | 49 | 1 | 0 | 1 |
| 100 | 3 | 1,100 / 900 | 45% | 2 | 41 | 1 | 1 | 1 |
| 101 | 3 | 1,000 / 1,000 | 50% | 2 | 35 | 1 | 1 | 1 |
| 102 | 3 | 2,000 / 0 | 0% | 1 | 50 | 17 | 0 | 0 |
| 103 | 3 | 1,200 / 800 | 40% | 1 | 29 | 17 | 1 | 1 |
| 104 | 3 | 1,300 / 700 | 35% | 2 | 37 | 17 | 0 | 0 |
| 105 | 3 | 1,700 / 300 | 15% | 2 | 22 | 10 | 1 | 0 |
| 106 | 3 | 1,200 / 800 | 40% | 2 | 20 | 10 | 0 | 0 |
| 107 | 3 | 200 / 1,800 | 90% | 2 | 22 | 13 | 1 | 1 |
| 108 | 3 | 2,000 / 0 | 0% | 2 | 49 | 13 | 0 | 0 |
| 109 | 3 | 0 / 2,000 | 100% | 2 | 19 | 13 | 1 | 0 |
| 110 | 3 | 1,500/ 500 | 25% | 2 | 50 | 3 | 1 | 1 |
| 111 | 3 | 2,000 / 0 | 0% | 2 | 48 | 3 | 0 | 1 |
| 112 | 3 | 1,000 / 1,000 | 50% | 2 | 30 | 7 | 1 | 0 |
| 113 | 3 | 800 / 1,200 | 60% | 2 | 53 | 18 | 1 | 1 |
| 114 | 3 | 1,000 / 1,000 | 50% | 2 | 42 | 7 | 1 | 0 |
| 115 | 3 | 1,100 / 90 | 45% | 2 | 24 | 6 | 1 | 0 |
| 116 | 3 | 400 / 1,600 | 80% | 2 | 32 | 6 | 1 | 0 |
| 117 | 3 | 500 / 1,500 | 75% | 2 | 33 | 6 | 1 | 1 |
| 118 | 3 | 1,000 / 1,000 | 50% | 2 | 51 | 14 | 1 | 0 |
| 119 | 3 | 800 / 1,200 | 60% | 1 | 63 | 6 | 1 | 1 |
| 120 | 3 | 900 / 1,100 | 55% | 1 | 56 | 6 | 1 | 1 |

**Table S4.**

Multilevel regression analysis with random intercept and fixed slopes predicting generosity in the Dictator Game.

|  | *b* | *se* | | *p* | | *95% CI LL* | *95%CI UL* | |
| --- | --- | --- | --- | --- | --- | --- | --- | --- |
| Intercept | 0.233 | | 0.043 | | 0.001* | 0.148 | 0.318 |  |
| NR | 0.045 | | 0.061 | | 0.459 | -0.075 | 0.164 |  |
| Pun | 0.051 | | 0.061 | | 0.395 | -0.068 | 0.171 |  |
| NR + Pun | 0.198 | | 0.061 | | 0.001 | 0.078 | 0.318 |  |

*NR = Needy recipient, Pun = supernatural punishment, NR + Pun = needy recipient combined with supernatural punishment*. * = *p* < .001.

**Table S5.**

Multilevel regression analysis with random intercept and random slopes predicting generosity in the Dictator Game

|  | b | *se* | | *p* | | *95% CI LL* | *95%CI UL* | |
| --- | --- | --- | --- | --- | --- | --- | --- | --- |
| Intercept | 0.224 | | 0.046 | | 0.001* | 0.133 | 0.317 |  |
| NR | 0.058 | | 0.063 | | 0.361 | -0.067 | 0.184 |  |
| Pun | 0.062 | | 0.063 | | 0.324 | -0.063 | 0.188 |  |
| NR + Pun | 0.202 | | 0.078 | | 0.010 | 0.049 | 0.356 |  |

*NR = Needy recipient, Pun = supernatural punishment, NR + Pun = needy recipient combined with supernatural punishment*. * = *p* < .001.

**S6: Syntax for the two analyses in SPSPP.**

MIXED GiveDG WITH needy_Recip Punish_dummy NR_and_P_dummy

/FIXED=needy_Recip Punish_dummy NR_and_P_dummy | SSTYPE(3)

/METHOD=REML

/print = solution

/RANDOM=INTERCEPT needy_Recip Punish_dummy NR_and_P_dummy | SUBJECT(village) COVTYPE(UNR).

MIXED GiveDG WITH needy_Recip Punish_dummy NR_and_P_dummy

/FIXED=needy_Recip Punish_dummy NR_and_P_dummy | SSTYPE(3)

/METHOD=REML

/print = solution

/RANDOM=INTERCEPT | SUBJECT(village) COVTYPE(UNR).

**Table S7.**

| ***Population Name*** | ***N*** | ***M*** | ***S. D.*** |
| --- | --- | --- | --- |
| *Henrich et al. (2014)* |  |  |  |
| Accra | 30 | 42 | 16.9 |
| Au | 30 | 41 | 19.6 |
| Dolgan/ Nganasan | 30 | 37 | 20.8 |
| Gusii | 25 | 33 | 5.4 |
| Hadza | 31 | 26 | 25.3 |
| Isanga | 30 | 36 | 18.3 |
| Maragoli | 25 | 35 | 17.1 |
| Orma | 26 | 42 | 15 |
| Samburu | 31 | 40 | 23.2 |
| Sanquianga | 30 | 47 | 15.6 |
| Shuar | 21 | 35 | 19.1 |
| Sursurunga | 30 | 41 | 18.6 |
| Tsimane' | 38 | 26 | 15.5 |
| U.S./ rural Missouri | 15 | 47 | 10.3 |
| Yasawa | 35 | 35 | 17.9 |
| *Marlowe (2004)* |  |  |  |
| Hadza | 43 | 20 | 16.2 |
| *Wiessner (2009)* |  |  |  |
| Ju/'hoansi | 53 | 20.13 | 15.96 |
| **Total** | **523** | **35.4782** |  |

**S8: Dataset**

Parties interested in the dataset used in the meta-analysis of Dictator Games (Engel 2011) may contact Christoph Engel at [engel@coll.mpg.de](mailto:engel@coll.mpg.de)
