# Supplementary figures and images for: Generosity among the Ik of Uganda
Source: Evol Hum Sci. 2020 May 14;2:e23. doi: 10.1017/ehs.2020.22 (PMC10427480; doi:10.1017/ehs.2020.22)

# Crls of all studies combined compared with CIs of each individual study

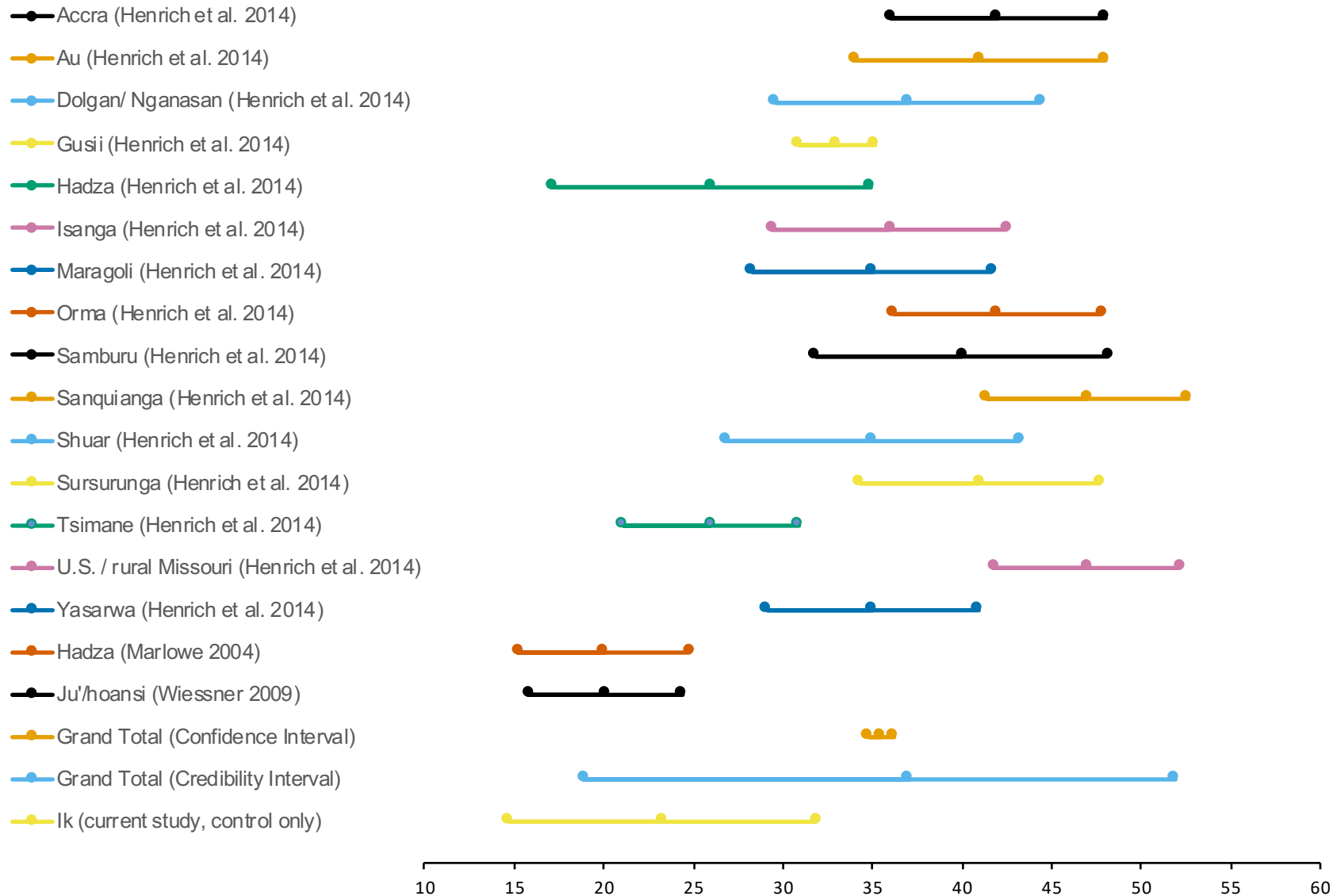

Supplement: Supplementary file 1 [file ehssup.zip › S2513843X20000225sup002.pdf]
